# The Cancer Stem Cell Marker CD133 Interacts with Plakoglobin and Controls Desmoglein-2 Protein Levels

**DOI:** 10.1371/journal.pone.0053710

**Published:** 2013-01-10

**Authors:** Ryo Koyama-Nasu, Rina Takahashi, Satoshi Yanagida, Yukiko Nasu-Nishimura, Masaaki Oyama, Hiroko Kozuka-Hata, Ryo Haruta, Emi Manabe, Akemi Hoshino-Okubo, Hiroko Omi, Nozomu Yanaihara, Aikou Okamoto, Tadao Tanaka, Tetsu Akiyama

**Affiliations:** 1 Laboratory of Molecular and Genetic Information, Institute of Molecular and Cellular Biosciences, The University of Tokyo, Bunkyo-ku, Tokyo, Japan; 2 Department of Obstetrics and Gynecology, The Jikei University School of Medicine, Minato-ku, Tokyo, Japan; 3 Medical Proteomics Laboratory, Institute of Medical Science, The University of Tokyo, Minato-ku, Tokyo, Japan; Northwestern University Feinberg School of Medicine, United States of America

## Abstract

The pentaspan membrane glycoprotein CD133 (also known as prominin-1) has been widely used as a marker for both cancer and normal stem cells. However, the function of CD133 has not been elucidated. Here we describe a cancer stem cell line established from clear cell carcinoma of the ovary (CCC) and show that CD133 interacts with plakoglobin (also known as γ-catenin), a desmosomal linker protein. We further demonstrate that knockdown of CD133 by RNA interference (RNAi) results in the downregulation of desmoglein-2, a desmosomal cadherin, and abrogates cell-cell adhesion and tumorigenicity of CCC stem cells. We speculate that CD133 may be a promising target for cancer chemotherapy.

## Introduction

Cancer stem cells are believed to have the capacity to proliferate and self-renew and to be responsible for tumorigenesis, metastasis and recurrence [1], [2]. The presence of cancer stem cells has been demonstrated in a variety of tumors [1]. In particular, glioblastoma stem cells have been extensively studied as they can be maintained in serum-free media that favor the growth of neural stem cells [3]. However, it is still difficult to maintain and expand cancer stem cells derived from other tissues *in vitro*. In the present study, we succeeded in establishing a cancer stem cell line from clear cell carcinoma of the ovary (CCC), which has the worst prognosis among epithelial ovarian cancers [4] and show that CD133 interacts with plakoglobin, controls desmoglein-2 protein levels and is required for cell-cell adhesion and tumorigenicity of CCC stem cells.

## Results and Discussion

**Figure 1:**
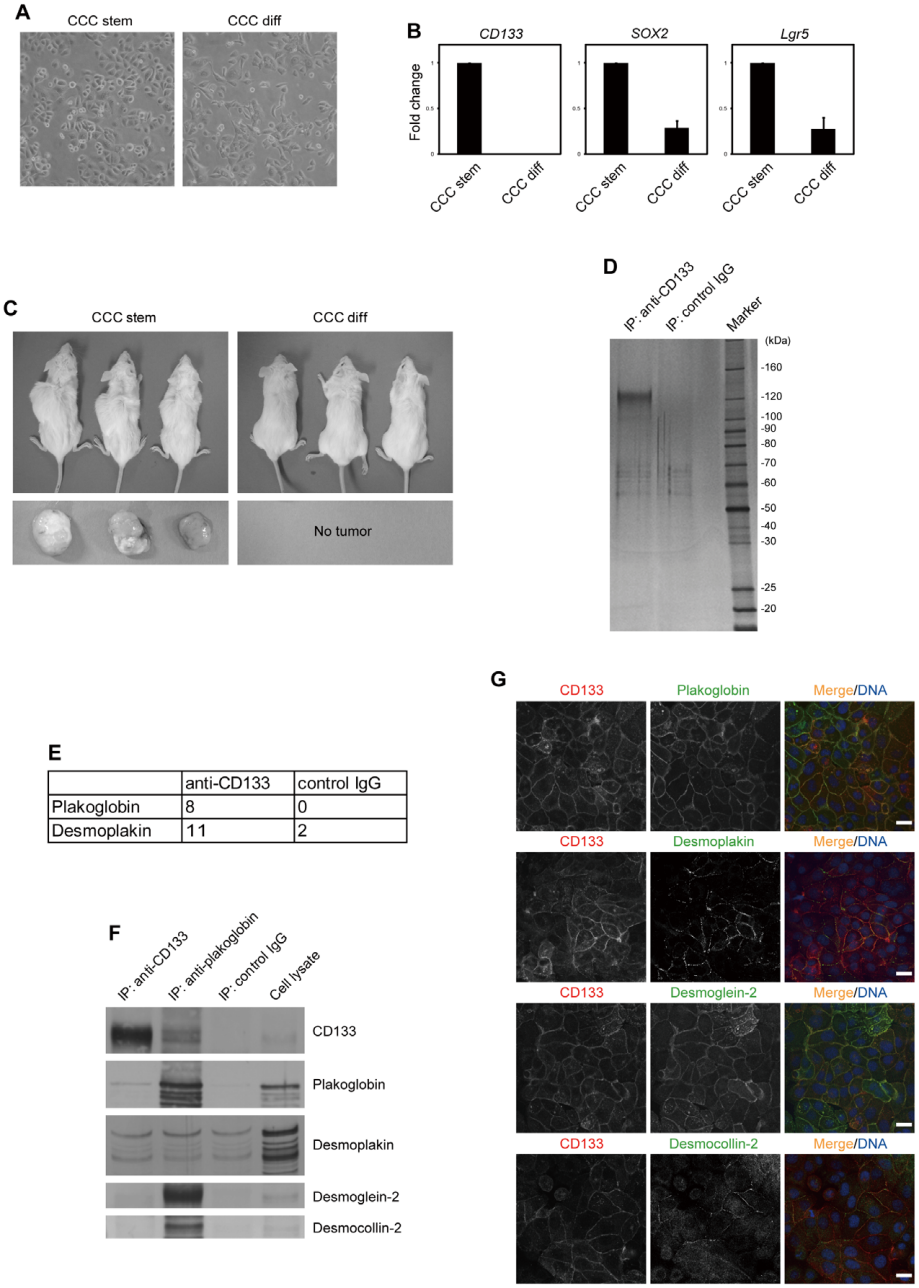
CD133 interacts with plakoglobin and localizes specifically to regions of cell-cell contact in CCC stem cells. (A) CCC stem and differentiated (diff) cells in culture. Phase contrast photographs are shown. (B) The mRNA levels of the indicated genes were evaluated by quantitative RT-PCR and shown as fold change over mRNA levels in CCC stem cells. Error bars represent the s.d. (*n* = 3). (C) CCC stem or differentiated cells were subcutaneously transplanted into NOG mice (*n* = 3). Seven months after transplantation, mice (upper) and tumors (lower) were photographed. (D) Elutes from immunopurified CD133 from the membrane fraction of CCC stem cells were resolved by SDS-PAGE and visualized by silver staining. (E) Desmosomal proteins identified by mass spectrometry. The numbers of unique peptides identified are shown. (F) Samples were prepared as described in (D) and immunoblotted with antibodies to the indicated proteins. (G) Co-localization of CD133 (red) with desmosomal proteins (green). CCC stem cells were immunostained with antibodies to the indicated proteins. TO-PRO-3 iodide was used for nuclear DNA staining (blue). Scale bars represent 20 µm.

We cultured CCC stem cells isolated from a patient diagnosed with CCC under serum-free conditions. Similar to glioblastoma stem cells [5], CCC stem cells grew exponentially on laminin-coated dishes under serum-free conditions (Fig. 1A and S1A). As reported previously for other cancers [3], [6], [7], CCC stem cells underwent differentiation when cultured in serum-containing medium (CCC differentiated cells): they exhibited a slight morphological change (Fig. 1A), and the expression levels of stem cell markers, such as *CD133* [8], *SOX2* [9] and *Lgr5* [10], were significantly reduced (Fig. 1B). When CCC stem cells were subcutaneously injected into immunocompromised mice, all mice developed tumors that were histopathologically similar to their original tumor (Fig. 1C and S1B). By contrast, none of the mice transplanted with CCC differentiated cells developed tumors, despite their capability to proliferate exponentially *in vitro* (Fig. 1C and S1A).

The expression of CD133 is strictly limited to a rare population of somatic and cancer stem cells [8]. It is therefore difficult to obtain sufficient numbers of cells to perform biochemical analysis of the CD133-containing protein complex. Taking advantage of the capability of CCC stem cells to grow exponentially and maintain high expression levels of CD133 *in vitro*, we set out to immunopurify the endogenous CD133 complex. CD133 was immunoprecipitated from the membrane fraction with anti-CD133 antibody and after confirmation by SDS-PAGE and silver staining, the immunoprecipitates were subjected to liquid chromatography-mass spectrometry (Fig. 1D). Among the co-purified proteins identified (Table S1), we focused our attention on plakoglobin and desmoplakin (Fig. 1E), since they are components of the desmosome, which mediates cell-cell adhesion [11]. Desmosomes are junctional complexes consisting of members of the cadherin family of cell adhesion proteins and linking proteins that attach the cell surface adhesion proteins to intracellular keratin cytoskeletal filaments. Plakoglobin and desmoplakin function as the main desmosomal linking proteins.

We confirmed the ability of CD133 to interact with plakoglobin by *in vivo* pull-down assays. When a lysate from CCC stem cells was subjected to immunoprecipitation with anti-CD133 antibody, followed by immunoblotting with anti-plakoglobin antibody, plakoglobin was found to have co-immunoprecipitated with CD133 (Fig. 1F). Plakoglobin was not detected when control IgG was used for immunoprecipitation. However, our *in vitro* pull-down assays failed to detect co-precipitation of plakoglobin with fragments containing individual cytoplasmic domains of CD133 (data not shown). This may be because the membrane topology of CD133 is important for its association with plakoglobin. Alternatively, CD133 may not be directly associated with plakoglobin. Results with desmoplakin were inconclusive, as it co-precipitated with either the anti-CD133 antibody or control IgG under our experimental conditions.

**Figure 2:**
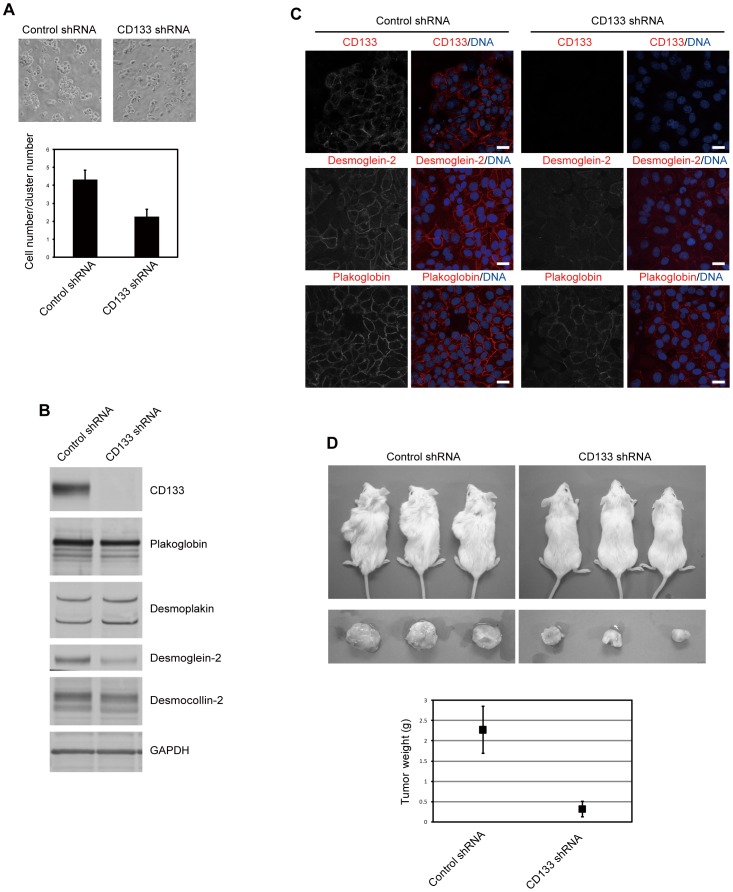
CD133 controls cell-cell adhesion and is required for tumorigenicity of CCC stem cells. (A) CCC stem cells were infected with a lentivirus expressing an shRNA targeting CD133. Cells were subjected to mechanical stress by pipetting in PBS containing 1 mM CaCl_2_ and 0.5 mM MgCl_2_. Representative images are shown (upper). The bar graph represents the ratio of cell number/cluster number (lower). Error bars represent the s.d. (*n* = 3). *p* = 0.011 with comparison to control shRNA by t test. (B) CCC stem cells were treated as described in (A). Cell lysates were subjected to immunoblotting with antibodies to the indicated proteins. (C) CCC stem cells were treated as described in (A). Cells were immunostained with antibodies to the indicated proteins (red). TO-PRO-3 iodide was used for nuclear DNA staining (blue). Scale bars represent 20 µm. (D) CCC stem cells were treated as described in (A). Cells were subcutaneously transplanted into immunocompromised mice (*n* = 3). Eleven months after transplantation, mice (upper) and tumors (middle) were photographed. The bar graph represents tumor weight (lower). Error bars represent the s.d. (*n* = 3). *p* = 0.007 with comparison to control shRNA by t test.

Immunohistochemical analysis of CCC stem cells revealed that CD133 and plakoglobin co-localized within regions of cell-cell contact (Fig. 1G). CD133 staining was not detected when cells were infected with a lentivirus expressing an shRNA targeting CD133 (Fig. 2C), indicating the specificity of anti-CD133 antibody. Desmoplakin was found to partially co-localize with CD133 (Fig. 1G).

We performed immunohistochemical analysis of desmoglein-2 and desmocollin-2, two desmosomal cadherins that are expressed in CCC stem cells. We found that these proteins also co-localized with CD133 (Fig. 1G). In particular, CD133 and desmoglein-2 had very similar distribution patterns. However, neither desmoglein-2 nor desmocollin-2 could be detected in CD133 immunoprecipitates, indicating that they are not physically associated (Fig. 1F). By contrast, plakoglobin immunoprecipitates were found to contain CD133 as well as desmosomal cadherins (Fig. 1F). Altogether, these results suggest that CD133 interacts with plakoglobin but not with the desmosomal protein complex containing desmoglein-2 and desmocollin-2.

We next studied the role of CD133 in the regulation of cell-cell adhesion. We observed that CCC stem cells could not be readily dispersed by pipetting. However, when cells were infected with a lentivirus expressing an shRNA targeting CD133, the cells could be dispersed by pipetting (Fig. 2A). Moreover, hanging drop cell aggregation assays demonstrated that CD133 knockdown cells did not aggregate tightly and could be dispersed by pipetting (Fig. S2). Thus, CD133 may be important for the adhesion of CCC stem cells. To elucidate the molecular mechanism underlying this decrease in cell-cell adhesion, we examined the expression levels of the desmosomal proteins. Immunoblotting and RT-PCR analyses revealed that knockdown of CD133 resulted in a decrease in the levels of desmoglein-2 protein, but not *desmoglein-2* mRNA (Fig. 2B and S3A), a result that was confirmed by immunohistochemistry (Fig. 2C).

Knockdown of CD133 using a distinct shRNA also resulted in downregulation of desmoglein-2 (Fig. S3B). Similar results were obtained with the human intestinal epithelial cell line Caco-2, which also expresses high levels of CD133 [12] (Fig. S3C). In addition, knockdown of CD133 led to a slightly diffused localization of plakoglobin (Fig. 2C). Furthermore, we found that knockdown of plakoglobin resulted in a decrease in the levels of desmoglein-2 protein (Fig. S3D, E). Consistent with these results, hanging drop cell aggregation assays revealed that knockdown of desmoglein-2 resulted in a decrease in adhesion of CCC cells (Fig. S2). These results suggest that CD133 is required for the stability and proper localization of desmosomal proteins.

CD133 is widely used to isolate a variety of cancer stem cells, including CCC of the ovary [8], [13]. However, its functional contribution to tumorigenesis has been unclear. Cell-cell adhesion is an inherent characteristic of solid tumors, and several reports have suggested that desmoglein-2 is essential for the tumorigenicity of several epithelial tumors [14]. Thus, we examined the potential role of CD133 in the tumorigenicity of CCC stem cells using an shRNA-encoding lentivirus to stably knockdown CD133 expression. Soft-agar colony-forming assays revealed that knockdown of CD133 resulted in a decrease in the colony-forming ability of CCC stem cells (Fig. S4). In addition, knockdown of desmoglein-2 also reduced colony formation. When the CD133-knockdown cells were subcutaneously injected into immunocompromised mice, they grew at a significantly reduced rate compared to the control cells (Fig. 2D). This result suggests that CD133 is required for the tumorigenicity of CCC stem cells.

In this report, we demonstrated that CD133 interacts with plakoglobin and controls cell-cell adhesion in CCC stem cells. Of particular interest is the fact that knockdown of CD133 in CCC stem cells caused a reduction in the levels of desmoglein-2. The mechanism by which the CD133-plakoglobin complex stabilizes desmoglein-2 remains to be investigated. In hematopoietic stem cells, CD133 is known to be enriched at the sites of contact with osteoblasts [15]. Thus, CD133 may function in both cancer and normal stem cells as a regulator of cell-cell interactions. We further showed that CD133 is important for the tumorigenicity of CCC stem cells. This finding is consistent with previous reports showing that desmoglein-2 is involved in tumorigenesis [14]. It is therefore intriguing to speculate that CD133 and/or desmoglein-2 may be therapeutic targets for CD133-positive epithelial cancer stem cells.

## Materials and Methods

### Tumor Specimen and Cell Cultures

The study was approved by the Ethics Committee for Biomedical Research of the Jikei Institutional Review Board, The Jikei University School of Medicine, Tokyo, Japan. All patients provided written informed consent. Tumor sample classified as clear cell carcinoma of the ovary was obtained from a patient undergoing surgical treatment at the Jikei University Hospital. Tumors were washed, and mechanically and enzymatically dissociated into single cells. Tumor cells were cultured on laminin (Sigma)-coated dish in DMEM/F-12 medium (Life Technologies) containing B27 supplement (Life Technologies), EGF and FGF2 (20 ng/ml each; Wako Pure Chemical Industries). For *in vitro* differentiation, tumor cells were cultured in DMEM/F-12 medium (Life Technologies) containing 10% fetal bovine serum. 293FT and Caco-2 cells were cultured in DMEM (Nissui) containing 10% fetal bovine serum.

### Subcutaneous Xenografts

One week after lentivirus infection, 1×10^5^ cells were injected subcutaneously into 6-week-old NOG mice (Central Institute for Experimental Animals) (*n* = 3). Tumors were histologically analyzed after hematoxylin and eosin (HE) staining. This study was approved by Animal Ethics Committee, The University of Tokyo, Tokyo, Japan. All animal experimental protocols were performed in accordance with the politics of the Animal Ethics Committee, The University of Tokyo, Tokyo, Japan.

### Quantitative RT-PCR

Total RNA was extracted using NucleoSpin RNA Clean-up kit (Takara) and reverse-transcribed into cDNA using PrimeScript RT Master Mix (Takara). Real-time PCR was performed using LightCycler480 SYBR Green I Master and a LightCycler480 Instrument (Roche). Results were normalized with the detected value for *glyceraldehyde-3-phosphate dehydrogenase (GAPDH)*. Primers used in real-time PCR were as follows: *GAPDH* forward (5′-GCACCGTCAAGGCTGAGAAC-3′), *GAPDH* reverse (5′-TGGTGAAGACGCCAGTGGA-3′); *CD133* forward (5′-AGTGGCATCGTGCAAACCTG-3′), *CD133* reverse (5′-CTCCGAATCCATTCGACGATAGTA-3′); *SOX2* forward (5′-TTGCTGCCTCTTTAAGACTAGGA-3′), *SOX2* reverse (5′-CTGGGGCTCAAACTTCTCTC-3′); *Lgr5* forward (5′-GATTTCCTGCTTGACTTTGAGG-3′), *Lgr5* reverse (5′-GCAGGTGTTCACAGGGTTTG-3′); *plakoglobin* forward (5′-GATCTTCCGGCTCAACACC-3′), *plakoglobin* reverse (5′-GATGTTCTCCACCGACGAGT-3′); *desmoglein-2* forward (5′-GGAAATTTTCAAGCTTTTGATGA-3′), *desmoglein-2* reverse (5′-CCACAGAGATCCAATTATCTCTATCTT-3′).

### Antibodies

Mouse monoclonal antibody (mAb) to CD133 (AC133) was obtained from Miltenyi Biotec. Mouse mAb to desmocollin-2/3 (7G6), desmoglein-2 (6D8) and α-tubulin were from Santa Cruz Biotechnology. Mouse mAb to plakoglobin was from BD Biosciences. Mouse mAb to GAPDH was from Millipore. Mouse mAb to desmoplakin (2Q400) was from Abcam and used for immunohistochemistry. Rabbit polyclonal antibody (pAb) to desmoplakin was from Abcam and used for immunoblotting.

### Immunoprecipitation and Immunoblotting

The membrane fraction of cells was lysed in lysis buffer [50 mM HEPES-NaOH pH 7.5, 150 mM NaCl, 1% NP40, 1 mM dithiothreitol and protease inhibitor cocktail (Roche)] (Fig. 1D). Alternatively, the membrane fraction was lysed in lysis buffer containing 1% digitonin instead of NP40 (Fig. 1F). Lysates were incubated with anti-CD133, anti-plakoglobin or control IgG1 immobilized to Activated CH-Sepharose 4B (GE Healthcare) for 4 h at 4°C. After five washes with lysis buffer, bound proteins were eluted with 100 mM Glycine-HCl and subjected to trypsin digestion. After desalted with ZipTip (C18; Millipore), the sample was subjected to liquid chromatography-mass spectrometry. For immunoblotting, the eluted proteins were fractionated by SDS-PAGE and transferred to a PVDF membrane (Immobilon-P, Millipore). The membrane was subjected to immunoblot analysis using alkaline phosphatase-conjugated anti-mouse or rabbit IgG (Promega) as secondary antibodies. Visualization was performed using the NBT/BCIP colorimetric substrate system (Promega).

### Mass Spectrometric Analysis and Protein Identification

Shotgun proteomic analyses were performed by a linear ion trap-orbitrap mass spectrometer (LTQ-Orbitrap Velos, Thermo Fisher Scientific) coupled with the nanoflow LC system (Dina-2A, KYA Technologies) as previously described [16]. Protein identification was conducted by searching MS and MS/MS data against the RefSeq (National Center for Biotechnology Information) human protein database (32,968 protein sequences as of Sep 12, 2011) using Mascot ver. 2.3.02 (Matrix Science). Methionine oxidation, protein N-terminal acetylation and pyro-glutamination for N-terminal glutamine were set as variable modifications. A maximum of two missed cleavages was allowed in our database search and the tolerance for mass deviation was set to 3 parts per million (ppm) for peptide masses and 0.8 Da for MS/MS peaks, respectively. Protein identification was based on the criterion of having at least one MS/MS data with Mascot scores that exceeded the thresholds (*p*<0.01).

### RNA Interference

The shRNA oligonucleotide sequences were as follows: CD133#1 (5′-GGAUACACCCUACUUACUAAA-3′), CD133#2 (5′-GCACUCUAUACCAAAGCGUCA-3′), desmoglein-2#1 (5′-GUUAGCGAGAGCAUGGAUAGA-3′), desmoglein-2#2 (5′-GCAUUAUCAGUAAUAAUUUGU-3′), plakoglobin (5′-GAGCAUGAUUCCCAUCAAUGA-3′).

### Lentivirus Production

Lentiviral vector (CS-RfA-CG) expressing an shRNA driven by the H1 promoter was transfected with the packaging vectors pCAG-HIV-gp and pCMV-VSV-G-RSV-Rev into 293FT cells using Lipofectamine 2000 Transfection Reagent (Life Technologies). All plasmids were kindly provided by H. Miyoshi (RIKEN BioResource Center, Japan). Viral supernatant was purified by ultracentrifugation at 25,000 rpm for 90 min (SW28 rotor, Beckman). Infection efficiency was monitored by green fluorescence protein (GFP) expression as it is driven by the CMV promoter.

### Immunohistochemistry

Cells were plated onto laminin**-**coated glass coverslips and fixed with ice-cold methanol. Cells were incubated with primary antibodies followed by incubation with secondary antibodies conjugated with Alexa 488 or 594 (Life Technologies). For CD133 visualization, cells were incubated with anti-CD133 antibody conjugated to R-phycoerythrin. TO-PRO-3 iodide (Life Technologies) was used for nuclear DNA staining. Cells were photographed with an LSM510 META laser scanning microscope (Carl Zeiss).

### Cell Dissociation Assay

5×10^4^ cells were seeded in laminin**-**coated 12-well plates. Cells were detached from culture plates with a cell scraper and passed 10 times through a 200-µl pipette tip in PBS containing 1 mM CaCl_2_ and 0.5 mM MgCl_2_. The images were captured by phase contrast microscopy. The extent of cell dissociation was represented by the ratio of cell number/cluster number.

### Hanging Drop Cell Aggregation Assay

Cells were trypsinized, washed with PBS and resuspended at 5×10^5^ cells per milliliter in medium. 7.5×10^3^ cells were suspended as hanging drops from the lid of culture dish and allowed to aggregate overnight. For trituration, cells were passed 10 times through a 200-µl pipette tip. The images were captured by phase contrast microscopy. The size of the particles was measured using ImageJ 1.46r software.

## Supporting Information

Figure S1 **Characterization of CCC stem cells.** (A) Proliferation kinetics of CCC stem cells. CCC stem and differentiated (diff) cells were cultured for the indicated times. The bar graph represents day (*x*-axis) and cell number (*y*-axis). (B) Histopathological analysis of tumor xenografts. HE staining of a CCC stem cells xenograft and patient tumor is shown.
(TIF)

Figure S2 **CD133 and desmoglein-2 are required for adhesion of CCC stem cells.** CCC stem cells were infected with a lentivirus expressing an shRNA targeting CD133 or desmoglein-2. Cells were seeded into hanging drop cultures and allowed to aggregate overnight. Before (-) and after (Trituration) cells were subjected to mechanical stress by pipetting, images were captured by phase contrast microscopy (upper). The bar graph represents mean particle size relative to cells expressing control shRNA (lower). Error bars represent the s.d. (*n* = 3). NS, not significant; *, *p*<0.05 by t test.
(TIF)

Figure S3 **CD133 and plakoglobin control the expression levels of desmoglein-2.** (A) CCC stem cells were infected with a lentivirus expressing an shRNA targeting CD133. The mRNA levels of the indicated genes were evaluated by quantitative RT-PCR and shown as fold change over mRNA levels in cells expressing control shRNA. Error bars represent the s.d. (*n* = 3). (B) CCC stem cells were infected with a lentivirus expressing an shRNA targeting CD133 (CD133 shRNA#2). Cell lysates were subjected to immunoblotting with antibodies to the indicated proteins. (C) Caco-2 cells were infected with a lentivirus expressing an shRNA targeting CD133. Cell lysates were subjected to immunoblotting with antibodies to the indicated proteins. (D) CCC stem cells were infected with a lentivirus expressing an shRNA targeting plakoglobin. Cell lysates were subjected to immunoblotting with antibodies to the indicated proteins. (E) CCC stem cells were treated as described in (D). The mRNA levels of the indicated genes were evaluated by quantitative RT-PCR and shown as fold change over mRNA levels in cells expressing control shRNA. Error bars represent the s.d. (*n* = 3).
(TIF)

Figure S4 **CD133 and demoglein-2 are required for anchorage-independent growth of CCC stem cells.** CCC stem cells were infected with a lentivirus expressing an shRNA targeting CD133 or desmoglein-2. Cells were seeded in soft-agar and cultured for 2 weeks. The bar graph represents the colony number relative to cells expressing control shRNA. Error bars represent the s.d. (*n* = 4). *, *p*<0.05 with comparison to control shRNA by t test.
(TIF)

Table S1 **Complete list of peptides identified in mass spectrometric analysis.**
(XLSX)
