# Supplementary figures and images for: The Cancer Stem Cell Marker CD133 Interacts with Plakoglobin and Controls Desmoglein-2 Protein Levels
Source: PLoS One. 2013 Jan 10;8(1):e53710. doi: 10.1371/journal.pone.0053710 (PMC3542344; doi:10.1371/journal.pone.0053710)

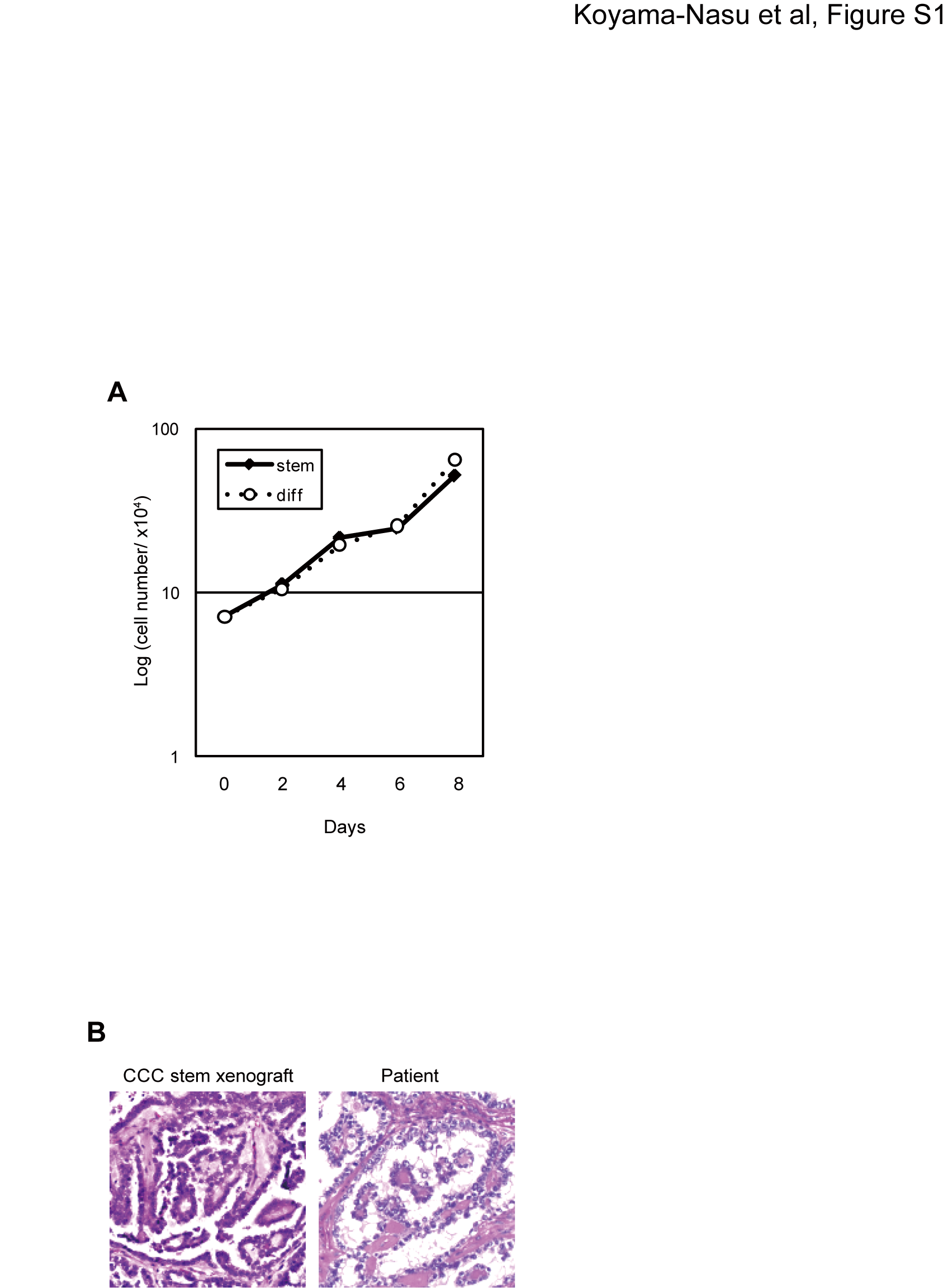

Supplement: Figure S1 — Characterization of CCC stem cells. (A) Proliferation kinetics of CCC stem cells. CCC stem and differentiated (diff) cells were cultured for the indicated times. The bar graph represents day (x-axis) and cell number (y-axis). (B) Histopathological analysis of tumor xenografts. HE staining of a CCC stem cells xenograft and patient tumor is shown. (TIF) [file pone.0053710.s001.tif]

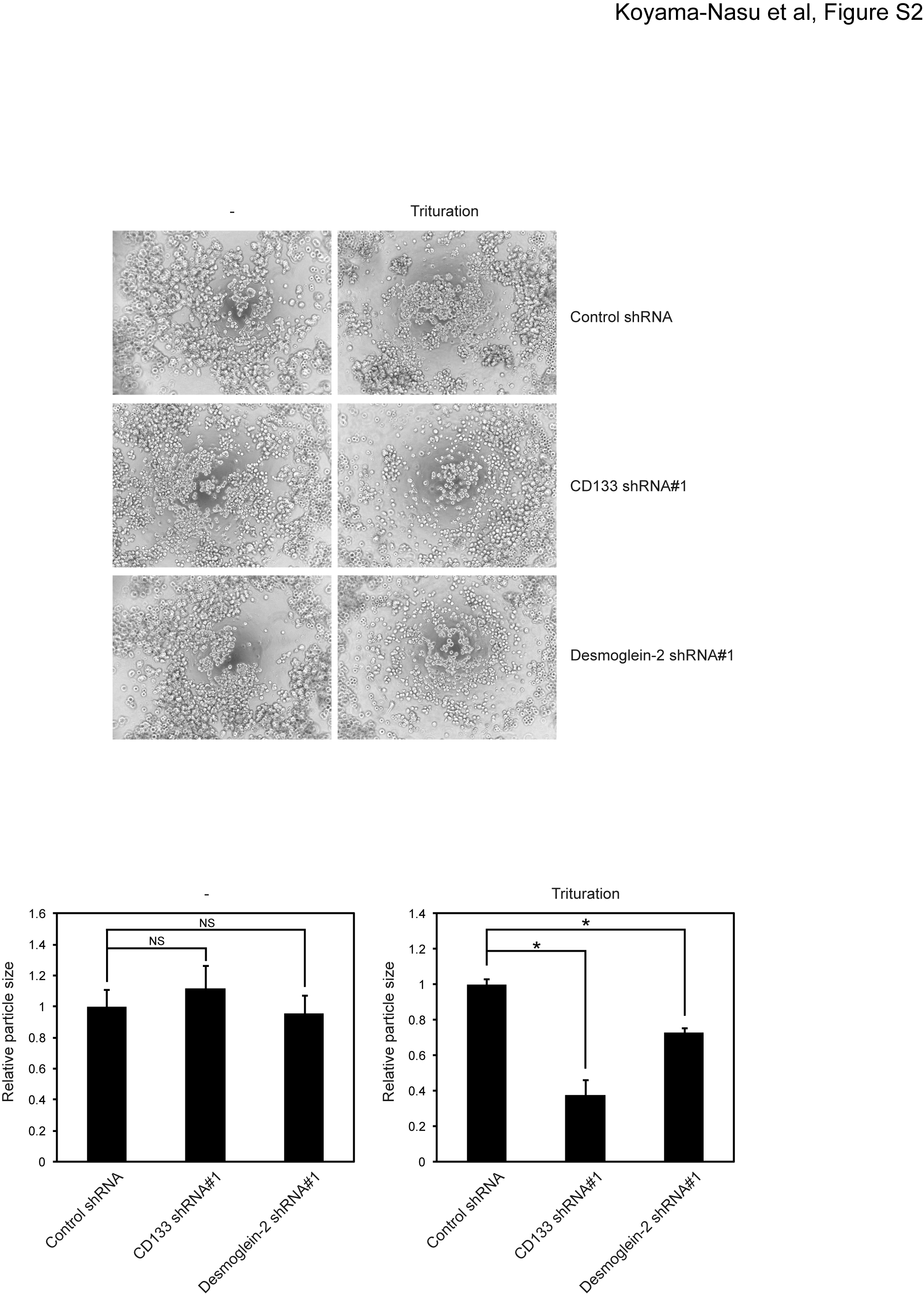

Supplement: Figure S2 — CD133 and desmoglein-2 are required for adhesion of CCC stem cells. CCC stem cells were infected with a lentivirus expressing an shRNA targeting CD133 or desmoglein-2. Cells were seeded into hanging drop cultures and allowed to aggregate overnight. Before (-) and after (Trituration) cells were subjected to mechanical stress by pipetting, images were captured by phase contrast microscopy (upper). The bar graph represents mean particle size relative to cells expressing control shRNA (lower). Error bars represent the s.d. (n = 3). NS, not significant; *, p<0.05 by t test. (TIF) [file pone.0053710.s002.tif]

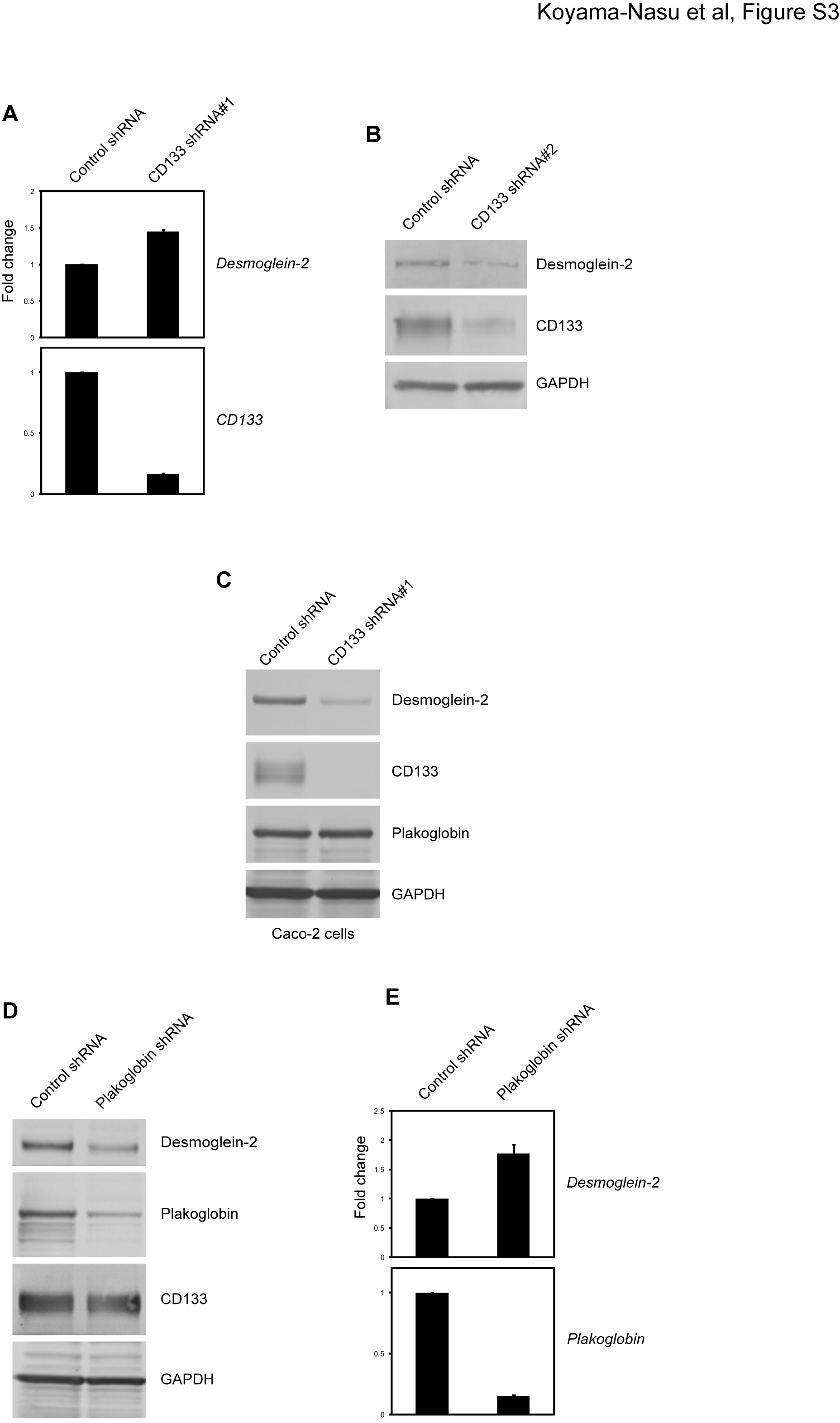

Supplement: Figure S3 — CD133 and plakoglobin control the expression levels of desmoglein-2. (A) CCC stem cells were infected with a lentivirus expressing an shRNA targeting CD133. The mRNA levels of the indicated genes were evaluated by quantitative RT-PCR and shown as fold change over mRNA levels in cells expressing control shRNA. Error bars represent the s.d. (n = 3). (B) CCC stem cells were infected with a lentivirus expressing an shRNA targeting CD133 (CD133 shRNA#2). Cell lysates were subjected to immunoblotting with antibodies to the indicated proteins. (C) Caco-2 cells were infected with a lentivirus expressing an shRNA targeting CD133. Cell lysates were subjected to immunoblotting with antibodies to the indicated proteins. (D) CCC stem cells were infected with a lentivirus expressing an shRNA targeting plakoglobin. Cell lysates were subjected to immunoblotting with antibodies to the indicated proteins. (E) CCC stem cells were treated as described in (D). The mRNA levels of the indicated genes were evaluated by quantitative RT-PCR and shown as fold change over mRNA levels in cells expressing control shRNA. Error bars represent the s.d. (n = 3). (TIF) [file pone.0053710.s003.tif]

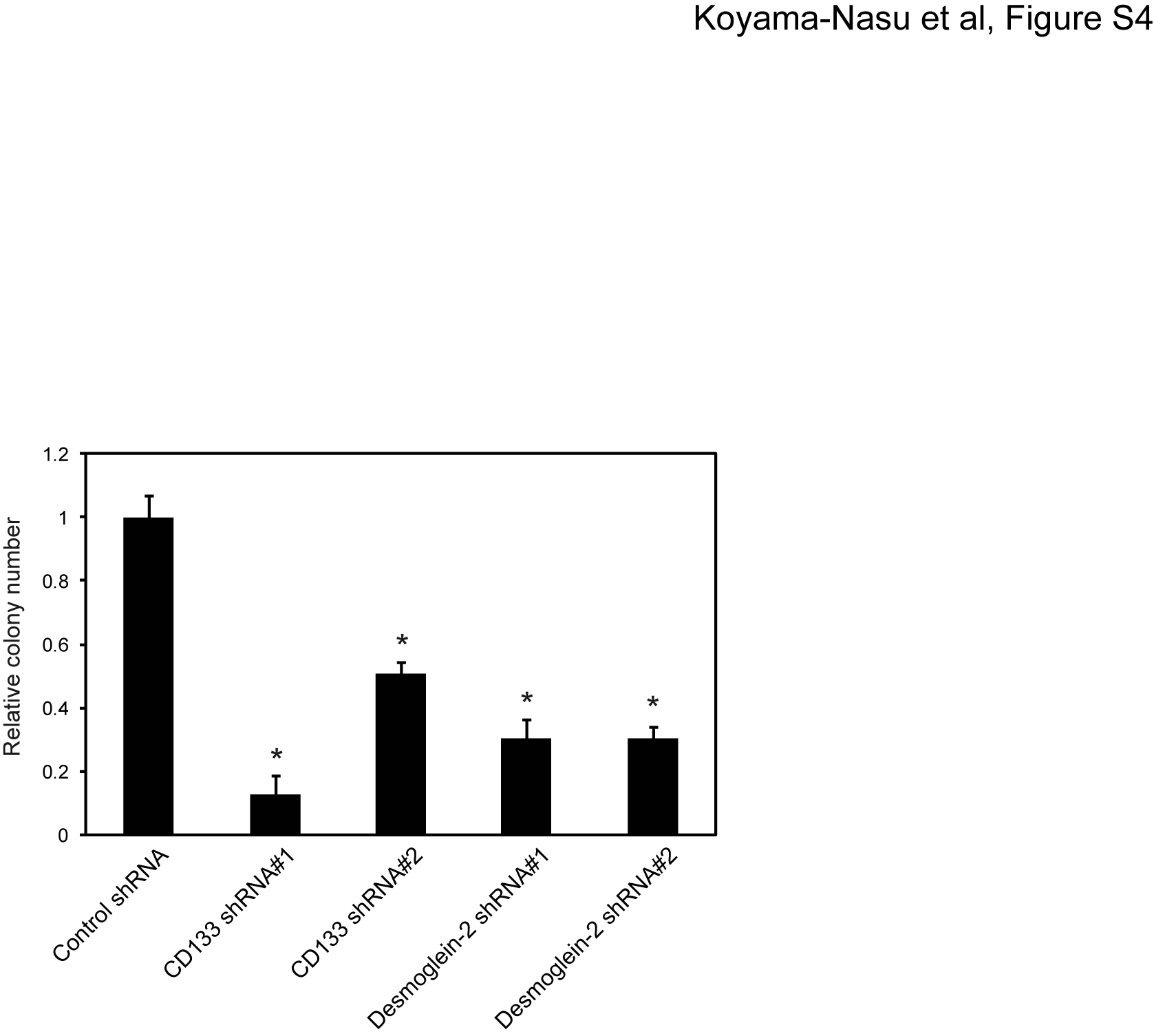

Supplement: Figure S4 — CD133 and demoglein-2 are required for anchorage-independent growth of CCC stem cells. CCC stem cells were infected with a lentivirus expressing an shRNA targeting CD133 or desmoglein-2. Cells were seeded in soft-agar and cultured for 2 weeks. The bar graph represents the colony number relative to cells expressing control shRNA. Error bars represent the s.d. (n = 4). *, p<0.05 with comparison to control shRNA by t test. (TIF) [file pone.0053710.s004.tif]
